# A Comparison of Arterial Blood Gas Data Between Open Esophagectomy and Thoracoscopic Esophagectomy

**DOI:** 10.14789/ejmj.JMJ24-0038-OA

**Published:** 2025-06-04

**Authors:** MARIKO AKIMOTO, DAIZOH SATOH, IZUMI KAWAGOE, YURI OOIZUMI-GOTO, CHIEKO MITAKA, TAKASHI HASHIMOTO, MASAKAZU HAYASHIDA

**Affiliations:** 1 Department of Anesthesiology and Pain Medicine, Juntendo University School of Medicine Graduate School of Medicine, Tokyo, Japan; 1 Department of Anesthesiology and Pain Medicine, Juntendo University School of Medicine Graduate School of Medicine, Tokyo, Japan; 2 Department of Esophageal and Gastroenterological Surgery, Juntendo University School of Medicine Graduate School of Medicine, Tokyo, Japan; 2 Department of Esophageal and Gastroenterological Surgery, Juntendo University School of Medicine Graduate School of Medicine, Tokyo, Japan; 3 Department of Anesthesiology and Pain Medicine, Juntendo University Nerima Hospital, Tokyo, Japan; 3 Department of Anesthesiology and Pain Medicine, Juntendo University Nerima Hospital, Tokyo, Japan

**Keywords:** thoracoscopic esophagectomy, arterial blood gas, one-lung ventilation

## Abstract

**Objectives:**

Minimally invasive thoracoscopic esophagectomy may result in superior post-operative outcomes compared to open esophagectomy. We compared arterial blood gas data during one-lung ventilation (OLV) between thoracoscopic esophagectomy and open esophagectomy.

**Design:**

37 patients undergoing thoracoscopic esophagectomy (Group E) and 38 patients undergoing open esophagectomy (Group O) were investigated.

**Methods:**

Arterial blood gas was analysed during two-lung ventilation (TLV) immediately before surgery (T1), during OLV for thoracic procedures (T2), during TLV for abdominal procedures (T3) and during spontaneous breathing immediately before extubation (T4).

**Results:**

Respiratory function data did not differ between the groups, even though the patients were older (*p* < 0.05) and the operative time was longer (*p* < 0.01) in Group E than in Group O. At T2, PaO_2_/F_I_O_2_ was lower (*p* < 0.01) and PaCO_2_ was higher (*p* < 0.01) in Group E than in Group O, although these variables did not differ between groups at T1 or T4. Post operative data showed shorter ICU (*p* < 0.01) and hospital stay (*p* < 0.05) in Group E than in Group O but showed no significant difference in the presence of complications.

**Conclusions:**

PaO_2_/F_I_O_2_ decreased and PaCO_2_ increased during OLV for thoracoscopic esophagectomy compared to open esophagectomy. Although thoracoscopic esophagectomy was inferior to open esophagectomy regarding gas exchange during OLV, patients in Group E required less ICU stay and less hospital stay than patients in Group O. The benefits of minimally invasive thoracoscopic esophagectomy may outweigh disadvantages regarding gas exchange during the surgery.

## Introduction

Thoracoscopic esophagectomy, introduced in 2017, is a less invasive surgery compared to conventional open esophagectomy via thoracotomy, and it may allow for enhanced post-operative recovery^1-3)^. We have largely shifted away from open esophagectomy at our hospital. In thoracoscopic esophagectomy, the prone position and pneumothorax using carbon dioxide (CO_2_) insufflation provide an adequate surgical field with a wide working space, regardless of whether two-lung ventilation (TLV) or one- lung ventilation (OLV) is used^4, 5)^. A double-lumen tube (DLT) or a bronchial blocker can be selected when OLV is employed, but a DLT can impede subcarinal lymph node dissection by limiting the mobility of the trachea and bronchus^6)^. Therefore, we utilise a combination of OLV with a bronchial blocker, pneumothorax and prone position.

One-lung ventilation in the prone position affords the surgeon an enhanced visualisation by increasing the working space. However, there are concerns regarding the effects of this approach on oxygenation and ventilation. Few studies have compared the effects of OLV with pneumothorax in the prone position for thoracoscopic esophagectomy on arterial blood gas data with those of OLV without pneumothorax for open esophagectomy in the lateral position.

## Methods

The study protocol was approved by the ethics committee of our hospital with a waiver of patients' written informed (Juntendo Clinical Research and Trial Center, H21-007) and registered at the University Hospital Medical Information Network (UMIN 000043544).

We retrospectively studied patients who underwent open and thoracoscopic esophagectomy for esophageal cancer between January 2017 and May 2021. Patients with an ASA-PS class III or more, those without complete arterial blood gas data sets and those who required conversion to an open procedure were excluded. We retrospectively investigated 75 patients comprising 37 patients who underwent thoracoscopic esophagectomy (Group E) and 38 patients who underwent open esophagectomy (Group O). Data on patients' intra-operative clinical characteristics were reviewed. The duration of surgery, anesthesia and OLV was collected. Intra-operative crystalloid and transfusion requirements, blood loss and urine output were reviewed as well.

Epidural anaesthesia was used in all patients. The epidural catheter was inserted between T6 and T10 before the induction of general anesthesia. All patients were induced with propofol and remifentanil. After muscle relaxation was achieved with rocuronium, endotracheal intubation was performed using a wire-reinforced tube (Shiley^TM^, EndoTracheal Tube Reinforced, Covidien, USA). General anesthesia was maintained with desflurane, sevoflurane, or propofol and remifentanil. Epidural anesthesia was maintained with levobupivacaine or ropivacaine combined with morphine. OLV during the thoracic procedure can be achieved by using either a double lumen tube or a bronchial blocker. In this study, OLV was achieved by using a bronchial blocker (Coopdech Endobronchial Blocker Tube^TM^, Diaken Medical, Japan). During OLV, a pressure-controlled or volume-controlled ventilation with a tidal volume of 5-8 mL/kg, peak inspiratory pressure below 30 cm H_2_O, respiratory rate of 10-16 breaths/min, fraction of inspiratory oxygen (F_I_O_2_) of 0.4-1.0 and positive end-expiratory pressure of 4 mmHg were applied to prevent hypoxaemia and hypercapnia.

In Group O, OLV without pneumothorax was performed, while the patients were placed in the left lateral decubitus position for open esophagectomy. The patients were placed in the supine position for the open abdominal approach and cervical portions of the operation, and TLV was resumed. In Group E, patients were placed in the prone position tilted slightly to the left, and OLV with artificial pneumothorax was maintained using 8 mmHg of CO_2_ insufflation. After the completion of the thoracic procedure, the patients were placed in supine lithotomy with a slightly head-up position, and TLV was resumed for the laparoscopic abdominal approach and cervical portions of the operation.

Arterial blood gas was analysed at the following four time points: during TLV immediately before surgery (T1), during OLV for thoracic procedures (T2), during TLV for abdominal procedures (T3) and during spontaneous breathing immediately before extubation (T4). Arterial blood gas for T2 and T3 were obtained after at least 10 minutes from the start of OLV and TLV, respectively. The F_I_O_2_ at the time of each blood gas analysis was available in the electronic medical record, and the ratio of partial pressure of oxygen (PaO_2_) to F_I_O_2_ (PaO_2_/F_I_O_2_) was calculated.

Patients were extubated in the operating room after certain criteria had been met: no atelectasis on chest radiograph, resumption of regular spontaneous breathing, recovery of consciousness and cough reflex and a respiratory rate between 10 and 25 breaths/min, a PaO_2_/F_I_O_2_ > 300 mmHg, no copious airway secretions, heart rate of less than 120 beats/min, systolic blood pressure between 90 and 160 mmHg not requiring inotropes or vasoactive medications, no clinically relevant arrhythmias and normal body temperature ≥ 36°C.

Complications were classified as either lung- related or others. Grade I and II complications according to the Clavien-Dindo classification^7)^ were classified as minor complications while Grade III and above were classified as major complications.

## Statistical analysis

Data are presented as the median (range) or the number (%). In the figures, PaO_2_ and PaCO_2_ are shown as the mean ± SD to simplify data presentation. All statistical analyses were performed using JMP pro16^TEM^ (SAS Institute Japan, Japan). The two groups were compared using the Mann-Whitney *U* test or the chi-square test according to data type. Changes in arterial blood gas data within a group were analysed with the Friedman test followed by the Scheffe multiple comparison test. A *p*-value < 0.05 was considered statistically significant.

## Results

Clinical characteristics of both groups are summarised in Table 1. There were no significant differences in the height, weight, body mass index, sex, smoking status, or preoperative pulmonary function test variables between groups. Patients were older in Group E than in Group O (*p* < 0.05). Durations of surgery and anaesthesia were longer in Group E than in Group O (*p* < 0.05), although there was no significant difference in the duration of OLV. Intra-operative blood loss was less in Group E than in Group O (*p* < 0.001). There were no significant differences between groups with respect to crystalloid or transfusion requirements or urine output. Post operative data showed shorter ICU (*p* < 0.01) and hospital stay (*p* < 0.05) in Group E than in Group O but showed no significant difference in the presence of complications. Minor lung- related complications included pleural effusion and aspiration pneumonia in both groups and a case of chylothorax in Group O. Major lung-related complications in Group O were one case of hypoxia at the end of surgery that required ventilation in the ICU until post operative day 1 and another case of respiratory failure caused by pyothorax that required drainage treatment. Minor complications that were not lung-related were minor leaks and a case of surgery site infection in Group E. Major complications that were not lung-related in Group O were a case of cerebral infarction and a case of suture failure that required drainage treatment. Major complications that were not lung-related in Group E were a case of cerebral infarction and a case of suture failure that required re-surgery.

**Figure 1:**
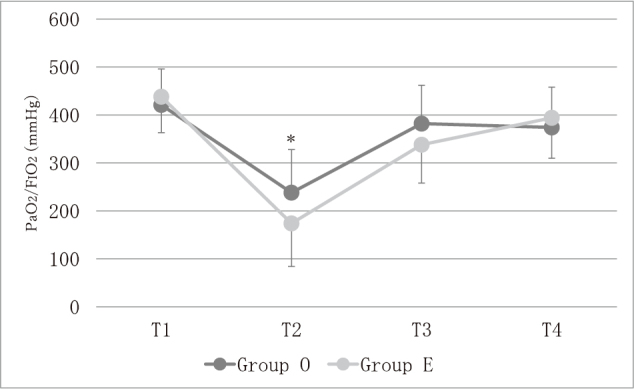
PaO_2_/F_I_O_2_ changes T1: before the operation in two-lung-ventilation, T2: during one-lung ventilation, T3: during two-lung-ventilation of the abdominal surgery, T4: at the end of the operation in spontaneous breathing before extubation, Group O: open esophagectomy group, Group E: thoracoscopic esophagectomy group ＊ *p* < 0.01, Group O *vs.* Group E

Changes in PaO_2_/F_I_O_2_ are shown in Figure 1. Although PaO_2_/F_I_O_2_ did not differ between groups at T1 (438 [250-682] *vs.* 421 [290-593]), at T3 (338 [193-445] *vs.* 382 [236-552]), or at T4 (394 [250-514] *vs.* 374 [140-484]), PaO_2_/F_I_O_2_ at T2 was lower in Group E than in Group O (174 [86-308] *vs.* 238 [70-410], *p* < 0.01) (Figure 1). In Group E and Group O, PaO_2_/F_I_O_2_ at T2 was lower than PaO_2_/F_I_O_2_ at T1, T3 and T4 (*p* < 0.001) (Figure 1).

**Figure 2:**
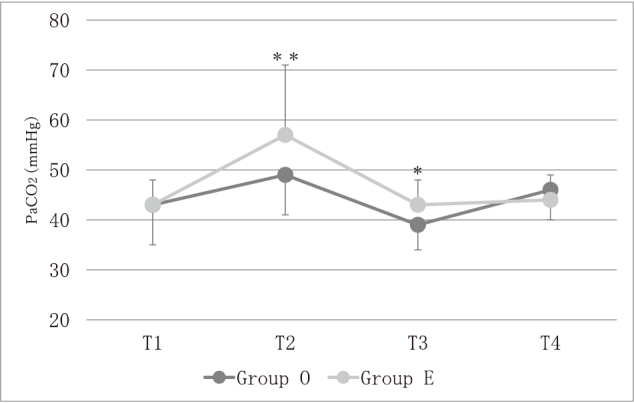
PaCO_2_ changes T1: before the operation in two-lung-ventilation, T2: during one-lung ventilation, T3: during two-lung-ventilation of the abdominal surgery, T4: at the end of the operation in spontaneous breathing before extubation, Group O: open esophagectomy group, Group E: thoracoscopic esophagectomy group ＊＊ *p* < 0.001, Group O vs. Group E ＊ *p* < 0.01, Group O vs. Group E

Changes in arterial partial pressure of CO_2_ (PaCO_2_) are shown in Figure 2. Although PaCO_2_ did not differ significantly between groups at T1 (43 [34-55] mmHg *vs.* 43 [31-63] mmHg) or at T4 (44 [35-56] mmHg *vs.* 46 [28-62] mmHg), PaCO_2_ was higher in Group E than in Group O at T2 (57 [30-94] mmHg *vs.* 49 [38-78] mmHg, *p* < 0.001) and at T3 (43 [33-58] mmHg *vs.* 39 [28-52] mmHg, *p* < 0.01). At T2, hypercapnia of PaCO_2_ more than 45mmHg were seen in 25 patients in Group E and in 30 patients in Group O, but there was no significant difference between the two groups (p = 0.13). At T3, 4 patients and 10 patients experienced hypercapnia in Group O and in Group E, respectively, but there was no significant difference between the groups (p = 0.06). In Group E, PaCO_2_ at T2 was higher than PaCO_2_ at T1, T3 and T4 (*p* < 0.001) (Figure 2). In Group O, PaCO_2_ at T2 was higher than PaCO_2_ at T1 and T3 (*p* < 0.001) and tended to be higher than that at T4 (p = 0.052) (Figure 2).

Five patients in Group E experienced an increase in PaCO_2_ above 70 mmHg during OLV with pneumothorax. In these patients, PaCO_2_ was reduced by decreasing CO_2_ insufflation pressure without requiring resumption of TLV. Two patients in Group O could not be extubated in the operating room because of a PaO_2_/F_I_O_2_ less than 300 mmHg.

**Table 1:** Patient demographic and perioperative data

	Group O (*n* = 38)	Group E (*n* = 37)	*p*
Demographics			
Age (year)	67 (48-81)	70 (33-85)	0.026
Weight (kg)	59 (35-80)	57 (37-84)	0.333
Height (cm)	165 (148-179)	164 (145-178)	0.363
BMI (kg/m^2^)	21 (15-28)	22 (15-27)	0.368
Sex (M/F)	31/7	32/5	0.568
Smoking status (Brinkman Index)	620 (0-1680)	570 (0-2700)	0.665
Vital Capacity (mL)	3591 (2260-4970)	3530 (1700-5640)	0.983
Percent Vital Capacity (%)	102 (74-126)	104(76-134)	0.880
Forced Expiratory Volume in 1second (mL)	25741460-3910)	2471(1470-4630)	0.578
FEV1/FVC (%)	74 (1470-4630)	74 (54-96)	0.841
Time (min)			
Operation	496 (243-894)	540 (346-663)	0.005
Anesthesia	591 (347-987)	625 (408-758)	0.014
One-lung-ventilation	239(126-389)	246 (102-377)	0.433
Total amount (ml)			
Infusion	5904 (2820-11370)	5789 (3160-9710)	0.875
Bleeding	499 (90-1150)	186(15-760)	< 0.001
Urine Volume	850(230-2400)	990 (190-3650)	0.512
Anesthesia data			
Sedation method (Desflurane/Sevoflurane/Propofol)	31/6/1	34/3/0	0.35
Epidural analgesia (Levobupivacaine/Ropivacaine)	22/16	17/20	0.31
Epidural site (Th6-7/Th7-8/Th8-9/Th9-10)	0/19/10/8	1/20/13/3	0.31
Post operative data			
ICU stay (days)	8 (5-16)	5 (2-8)	< 0.0001
Hospital stay (days)	20 (13-61)	17 (8-93)	0.0294
Complications			
Minor. lung-related	5	3	0.48
Major, lung-related	2	0	0.16
Minor, others	4	8	0.19
Major, others	2	2	0.98

group O: open esophagectomy group, group E: thoracoscopic esophagectomy group, FEV1: Forced Expiratory Volume in 1second, FVC: Forced vital capacity Data are median (range)

## Discussion

In this study, we found that PaO_2_/F_I_O_2_ was lower and PaCO_2_ was higher during OLV with artificial pneumothorax for thoracoscopic esophagectomy in the prone position than during OLV without pneumothorax for open thoracic esophagectomy in the lateral position. However, PaO_2_/F_I_O_2_ and PaCO_2_ during TLV before surgery and during spontaneous breathing after surgery did not differ between these surgical procedures.

Radical esophagectomy comprises thoracic, abdominal and cervical procedures. OLV using either a DLT or a bronchial blocker has commonly been used to provide access to the esophagus during the thoracic procedure in open esophagectomy. At our hospital, we use a bronchial blocker to achieve OLV for both open and thoracoscopic esophagectomies.

There are different airway management approaches for thoracoscopic esophagectomy^4-6, 8)^. Artificial pneumothorax with CO_2_ alone or a combination of pneumothorax and OLV using a DLT or bronchial blocker can be utilised to provide adequate exposure of the esophagus during the thoracic portion of the operation^4-6, 8)^. There are reports which suggest that a bronchial blocker is inferior to a DLT in its ability to achieve total collapse of the non-ventilated lung^9)^. An increased shunt fraction secondary to atelectasis can impair oxygenation during OLV with a bronchial blocker in both thoracoscopic and open surgery. In our practice, we could achieve total lung collapse using a bronchial blocker in both groups by positioning it just distal to the origin of the right mainstem bronchus. Total lung collapse with a bronchial blocker was achieved more slowly than a DLT.

Two studies suggest superior oxygenation in the prone position for thoracoscopic esophagectomy employing artificial pneumothorax than in the lateral position for open esophagectomy^5, 9)^. The difference was attributed to enhanced preservation of functional residual capacities (FRC) and ventilation/perfusion matching resulting from the prone position^5, 9-11)^. However, these two studies were different from our study in terms of study design. One study employed TLV with artificial pneumothorax in the prone position for thoracoscopic esophagectomy and compared it with historical controls of open thoracic surgery using OLV in the lateral position^5)^. The second study compared oxygenation during OLV using a bronchial blocker combined with pneumothorax in only nine patients undergoing thoracoscopic esophagectomy in the prone position with oxygenation during OLV using a DLT in only nine patients undergoing open esophagectomy in the lateral position^9)^. Additionally, FRC in the prone position does not differ significantly from that in the lateral position during general anesthesia^12)^. Previous studies have reported that the initiation of OLV with pneumothorax causes significant reductions in static lung compliance and PaO_2_ and increases PaCO_2_ during thoracoscopic esophagectomy^11)^. Our study demonstrated decreases in PaO_2_/F_I_O_2_ and increases in PaCO_2_ following OLV with artificial pneumothorax in the prone position compared with OLV alone in the lateral position.

The lower PaO_2_/F_I_O_2_ during OLV for thoracoscopic esophagectomy may be attributed to the reduced effect of gravity from prone positioning along with reduced static lung compliance^11, 13)^. In the lateral position, hypoxic pulmonary vasoconstriction and gravity reduce pulmonary blood flow (PBF) in the nondependent lung from 20% to 40% of total PBF^14)^. Lateral positioning during OLV permits gravity-induced redistribution of PBF to the dependent ventilated lung, and this can improve ventilation/perfusion mismatch. Conversely, prone positioning does not render such PBF redistribution^13)^. This may explain the position-related differences in oxygenation in our study. Theoretically, artificial pneumothorax with CO_2_ insufflation may impair oxygenation during OLV for thoracoscopic esophagectomy by increasing the alveolar CO_2_ partial pressure and thereby decreasing alveolar O_2_ partial pressure.

In our study, PaCO_2_ was higher during OLV for thoracoscopic esophagectomy. Elevated PaCO_2_ during thoracoscopy could result from alveolar hypoventilation, ventilation/perfusion mismatch and/or CO_2_ dissolution into the blood. As mentioned above, prone positioning does not permit correction of ventilation/perfusion mismatch by gravity-induced redistribution of PBF to the ventilated lung^13)^. Therefore, increased ventilation/perfusion mismatch is considered to contribute to higher PaCO_2_ during thoracoscopy. CO_2_ dissolution into the blood secondary to CO_2_ insufflation was considered to also contribute to higher PaCO_2_ during thoracoscopy^15)^. Severe hypercapnia during prone position and during OLV must be treated promptly for patient's safety. Decreasing the insufflation pressure and terminating OLV should be considered. As PaCO_2_ normalizes, minimizing PEEP could be considered for better surgical view. Resumption of OLV should be considered after PaCO_2_ returns to normal level and if surgical view with TLV and CO_2_ insufflation is insufficient. In our study, all five patients in Group E, who experienced elevated PaCO_2_ above 70 mmHg were successfully managed by decreasing insufflation pressure and did not require the resumption of TLV.

In our study, patients who underwent thoracoscopic surgery were older and had longer durations of anaesthesia and surgery, but intra-operative bleeding was significantly less and the surgical incisions were smaller. These factors may have contributed to better post-operative recovery, as patients who received thoracoscopic esophagectomy required less ICU stay and less hospital stay than patients who received open esophagectomy but showed no significant difference in the presence of complications. Two patients in Group O could not be extubated in the operating room, whereas there were no failed extubations in Group E. Therefore, minimally invasive esophagectomy may facilitate enhanced post-operative recovery. Furthermore, differences in intra-operative PaO_2_ and PaCO_2_ between the two groups disappeared when the patients were spontaneously breathing after the conclusion of surgery. Therefore, impaired oxygenation and CO_2_ elimination during OLV in thoracoscopy may be acceptable in exchange for the benefits of minimally invasive surgery.

## Limitations

This study has several limitations. Firstly, this study was not a prospective randomised trial. Secondly, we did not address patients' blood gas data after surgery. Thirdly, we did not study pulmonary compliance, functional residual capacity of the ventilated lung or patient hemodynamics during OLV. Further investigation is needed to corroborate our findings.

## Conclusions

There were significant decreases in PaO_2_/F_I_O_2_ and increases in PaCO_2_ during OLV for thoracoscopic esophagectomy performed in the prone position compared with open esophagectomy performed in the lateral position. However, differences between groups in PaO_2_/F_I_O_2_ and PaCO_2_ disappeared when the patients began to spontaneously breathe upon conclusion of surgery. Temporal disadvantages in gas exchange during OLV with pneumothorax for thoracoscopic esophagectomy seemed acceptable given the benefits of employing a minimally invasive approach to an otherwise invasive procedure.

## Funding

No funding was received.

## Author contributions

MH and DS designed and conceived this study. MA and YOG collected data. MA, DS, and CM analyzed and interpreted the results. MA drafted the manuscript. IK and TH supported statistical analyses. All authors read and approved the final manuscript.

## Conflicts of interest statement

The authors declare that there are no conflicts of interest.
